# Risk factors in the assessment of suppliers

**DOI:** 10.1371/journal.pone.0272157

**Published:** 2022-08-01

**Authors:** Maciej Urbaniak, Dominik Zimon, Peter Madzik, Eva Šírová

**Affiliations:** 1 Department of Logistics, Faculty of Management, University of Lódź, Łódź, Poland; 2 Department of Management Systems and Logistics, Rzeszow University of Technology, Rzeszow, Poland; 3 Department of Business Administration and Management, Technical University of Liberec, Liberec, Czech Republic; University of Catania, ITALY

## Abstract

The need to evaluate suppliers from the perspective of risk analysis by purchasing companies is increasing. Such evaluation of suppliers is conducted primarily by production companies with implemented quality (QMS), environmental (EMS), health and safety management systems (H&SMS), as well as Toyota Production System (TPS). This article aims to examine latent factors for suppliers’ evaluation and to describe the intensity of these factors by the implemented management system. The article provides the results of empirical research conducted with the computer-assisted telephone interviewing (CATI) technique in 151 medium and large manufacturing companies operating in Poland. The risk was classified into three main groups to deepen the research process: management system risks, environment risks, and process risks. This allowed for the formulation of some original conclusions. The results showed that companies implementing standardized management systems take the issue of risk analysis and management more seriously than organizations that do not implement such systems. The research also highlighted the differences in the perception of risk caused by implementing various management systems. The study also found that the industry and business profile specificity also affect the risk assessment in cooperation with suppliers.

## 1. Introduction

Enterprises are becoming more aware that building their competitive advantage also requires partner relations with suppliers [1–4]. Successful building of these relationships is based on the development of mutual trust and the continuous analysis of potential risks related to purchasing [5–8]. To reduce the scope and level of potential threats, companies conduct an initial and periodic assessment of suppliers from the perspective of risk analysis [9, 10]. This analysis considers the risks related to the products (such as quality defects, low level of innovation and technology, negative impact on the environment, response to complaints) and delivery performance (timeliness, flexibility, production capacity, communication problems with suppliers). When conducting a risk analysis, buyers also consider the possibility of increasing or reducing delivery costs and the financial situation of suppliers. For these reasons, suppliers’ preliminary assessment and selection play a particularly important role. This assessment and selection results should reduce the risk of future collaboration between customers and suppliers [11–13].

A preliminary assessment of suppliers is conducted by: the analysis of offers, self-assessment questionnaires, audits, and the testing of a batch of products. This assessment also includes examining potential partners’ economic and legal situations through due diligence [14, 15]. Such information on suppliers should reduce the risk associated with product deliveries and relations with future partners. Audits are particularly important in verifying the collected information [16]. During audits at suppliers, in particular, operational processes are assessed, such as customer service (accepting orders, handling complaints), research and development, production planning, production preparation, production, maintenance, product quality control, process quality control, packaging, storage, product shipment as well as after-sales service. When assessing suppliers through audits, particular attention is paid to the documentation used (procedures and instructions, as well as processes records).

Documented records of process and product control, personnel qualifications, workplace safety, and the environmental impact (reducing the consumption of raw materials and waste, reducing emissions, recycling, and the disposal methods used) are essential in this assessment during audits. In many industrial sectors (such as automotive, electromechanical, or chemical), suppliers are required to conduct risk analysis using FMEA (failure modes and effects analysis) for products and processes [17–19]. This analysis includes the determination of potential non-conformities, the probability of their occurrence, and the severity of possible errors for the company (occurrence of disruptions in processes, losses) and/or for customers. The preliminary evaluation results of suppliers are a condition for their qualification [20–22]. The positive status of this qualification may reduce the risk of cooperation with suppliers. By entering into a partnership with qualified suppliers, customers monitor the quality and timeliness of deliveries on an ongoing basis [23]. Customers also periodically conduct a comprehensive and multi-criteria supplier assessment [24, 25].

It is a truism to say that good cooperation with suppliers affects the effectiveness and efficiency of entire supply chains [26, 27]. Companies implement various management concepts to improve supplier collaboration and minimize threats and risks [28].

When reviewing the literature, there is a lack of research and studies on the impact of standardized management systems such as QMS, EMS, H&SMS, and TPS on the risk management process in cooperation with suppliers [26, 29]. It seems essential to research in this area, as these systems increasingly refer to the concept of risk management [30]. Dellana et al. [27] suggest that ISO 9001 provides a framework for risk management processes and collaboration with supply chain partners. This article aims to examine latent factors for suppliers’ evaluation and to describe the intensity of these factors by the implemented management system. With this in mind, the main aim of this research was decomposed into the following questions:

1. What is the perception of risk factors in evaluating suppliers?
2. Does the manufacturing companies’ implementation of management systems affect their assessment and perception of risk factors in cooperation with suppliers?
3. Which systems do entrepreneurs believe have the greatest impact in this regard?
4. Do company characteristics, such as the sector, size, or type of capital, affect the different assessment and perception of risk in relation to suppliers by manufacturing companies?

Answering these questions will allow for the writing of one of the first articles covering the scope of the impact of implementing multiple management systems on the risk assessment of suppliers.

## 2. Process improvement tools and risk assessment

The frequency of the periodic assessment depends on the intensity of purchasing processes and the risk associated with the supply of products [31, 32]. The technical quality of the products is of particular importance in this assessment. Such situations are events and crises that may disrupt the proper delivery of supplies (e.g., floods, tsunamis, hurricanes, earthquakes, fires, technological failures, catastrophes, sabotage, terrorism, strikes, and loss of commercial or financial credibility). The effective implementation of these plans should guarantee the continuity of the supply chain and ensure the security of supply and resistance to disruption [33–36].

An essential criterion for assessing suppliers is the flexibility of deliveries related to the possibility of changing the order in terms of terms, quantity, sequencing, or the type of assortments of products purchased [37, 38]. When assessing suppliers, enterprises increasingly consider the efficiency of communication processes [39]. Communication problems with suppliers may relate to errors in assortments and documentation confirming the delivery date and too long response time to complaints; in particular, enterprises with implemented quality, environmental, health, and safety management systems (QEH&SMS) consider the above criteria for assessing suppliers from the perspective of risk analysis. These systems are based on the concept of risk management [40–43]. Medina Serrano et al. [28] emphasize that implementing the ISO management standards allows enterprises to manage risk in contact with suppliers actively. Dellana et al. [27] suggest that QEH&SMS provides a framework for risk management processes and collaboration with supply chain partners. Buyers with implemented QESMS focus their requirements on suppliers, expecting them to improve products and processes [44]. When formulating these requirements for suppliers, companies that are customers also consider the concept of risk management [45]. The international management standards published by the International Organization for Standardization are currently based on this concept. Implementing this concept significantly impacts risk reduction and contributes to ensuring the continuity of processes by partners in the supply chain [46, 47].

Recently, many enterprises, especially international corporations, have assessed suppliers taking into account the concept of sustainability [48–54]. These companies evaluate their suppliers to meet the principles of business ethics and improve environmental impact [7, 55–57]. These rules are published in the supplier code of conduct [58, 59]. These codes are based on the principles of the Global Compact (relating to respect for human rights, ensuring labor standards, environmental protection, and anti-corruption). Enterprises expecting suppliers to implement the sustainability concept require them to achieve goals in the form of target indicators related to environmental protection (such as reducing the consumption of harmful substances and carbon dioxide emissions), improving product safety (reducing the number of manufacturing defects, customer complaints) and processes (reducing the risk of accidents or emergencies). Therefore it can be concluded that the supplier assessment considering the risk management concept is multi-criteria [60–62].

Multinationals often require their suppliers to report on product and process improvement regularly [63]. Information provided by suppliers through Performance Feedback Reports Cards relates to results in reducing costs, reducing product non-compliance, improving process efficiency and effectiveness indicators, reducing material/energy consumption, shortening process cycle times, and optimizing the use of production capacity [64–66]. Industrial buyers (especially Original Equipment Manufacturers) offer their suppliers special development programs to reduce the risk related to products and delivery [67, 68]. These programs are oriented towards the implementation of risk management concepts to reduce the level of quality defects and avoid delivery delays. Effective implementation of these programs allows both suppliers and recipients to improve the quality of products (reduce the level of non-compliance of products, introduce innovations, increase the level of environmental performance, reliability, safety), shorten process cycles, reduce the negative impact on the environment as well as reduce costs [69]. Many international concerns try to help local suppliers meet their stringent requirements by assisting them in consultations and training in QEH&SMS. Through development programs, purchasing companies try to educate their suppliers in operational improvement tools, such as Six Sigma, Toyota Production System, or Lean Management. These tools are implemented through joint projects with suppliers [70] (Saghiri & Wilding, 2021). The supplier development programs that are increasingly being offered include the implementation of the sustainability concept [71–73]. Sustainable supplier development programs ensure the safety of products and processes in supply chains, reduce the negative impact on the environment, improve working conditions, and promote ethical behavior in economic relations [74, 75].

## 3. Methodology

### 3.1 Survey instrument

A survey was chosen to cover the set research objective. Based on a literature review, 21 different types of risks associated with the management system were identified. These risks were variables that were developed into questions for respondents. A 5-point Likert scale was used, ranging from 1 (low importance of risk) to 5 (high importance of risk). The abbreviations of the variables shown in Table 1 were used in the results.

**Table 1:** Variables and their codes.

Variable	Measure	Code
Quality defects of products	Ordinal	QualDef
Assortment mistakes in deliveries	Ordinal	AsMist
Low level of environmental performance of products	Ordinal	EnvPerf
Threats to timely deliveries	Ordinal	TimDel
Low level of employee qualifications	Ordinal	EmpQual
Suppliers’ financial standing	Ordinal	SupFin
Low level of after-sales service	Ordinal	AftSal
Limited production capacity	Ordinal	ProdCap
Low level of product innovation	Ordinal	ProdInn
Problem with product identification	Ordinal	ProdIde
Errors in the delivery documentation	Ordinal	DocErr
Long order processing time	Ordinal	LonTim
No emergency delivery plans	Ordinal	EmePla
Technological problems	Ordinal	TechProb
Unjustified raising prices for products	Ordinal	RaisPri
Low level of supplier involvement in joint research and development	Ordinal	SupInvRes
Maladjustment of information systems in communication	Ordinal	MalaIS
Low level of supplier involvement to reducing operating costs	Ordinal	SupInvCos
Communication problems (related to the transfer of requirements and their confirmation by the supplier)	Ordinal	ComProb
Low level of delivery flexibility	Ordinal	DelFlex
Long response time to complaints	Ordinal	CompResp
Number of employees	Ordinal	Size
Implementation of QMS (yes/no)	Nominal	ISO9001
Implementation of EMS (yes/no)	Nominal	ISO14001
Implementation of H&SMS (yes/no)	Nominal	OHSAS
Implementation of Toyota Production System (Kaizen, 5S, TPM) (yes/no)	Nominal	TPM
Capital (domestic/foreign)	Nominal	Capital
Sector (Chemical/automotive/furniture/electromechanical)	Nominal	Sector

### 3.2 Sample selection and data collection

The subject of the research was to define the importance of supplier evaluation criteria from the perspective of risk analysis in the opinion of the surveyed production companies. The study was conducted between October and November 2019 using the Computer Assisted Telephone Interview (CATI) technique. The research covered 151 producers (employing over 49 people) who were suppliers for enterprises from the automotive, metal, chemical, and furniture manufacturing sectors operating in the Polish business-to-business (B2B) market. 39% of the surveyed economic entities were enterprises with foreign capital (including large international concerns with global activity). Supplier evaluation criteria related to risk were assigned a rank on a scale from one (low importance of risk) to five (high importance of risk). The study was commissioned by a specialized research agency that targeted companies registered in the Bisnode database, a business directory search platform. The research agency obtained informed consent from each survey participant and recorded telephone calls.

### 3.3 Used method and procedures

The data collected were tested for scale reliability. A Cronbach‘s alpha metric was used. At the same time, the relevance of the variables was tested. The level of Cronbach’s alpha was checked, removing individual variables. Non-response bias was assessed by sample distribution [76]. Samples were split into (n = 83) and late (n = 68) responses. A T-test of 10 random variables was performed to examine non-response bias [77].

Descriptive statistics were used for the basic examination of the results, supplemented by a graphical presentation. From the descriptive statistics, position measures, central tendency measures, of dispersion measures were used. A bivariant correlation analysis was used from the inference statistics, using Pearson‘s linear correlation coefficient. Latent relationships between variables were examined by exploratory correlation analysis. In this analysis, PCA (principal component analysis) was used as the extraction method, and for better results, the factor matrix was rotated using the Varimax method. The standardized Z-score was calculated using a linear regression model. A T-test was used to verify the statistical significance of the differences–the data were divided into groups according to whether or not the examined management system was implemented.

## 4. Results

**Fig 1:**
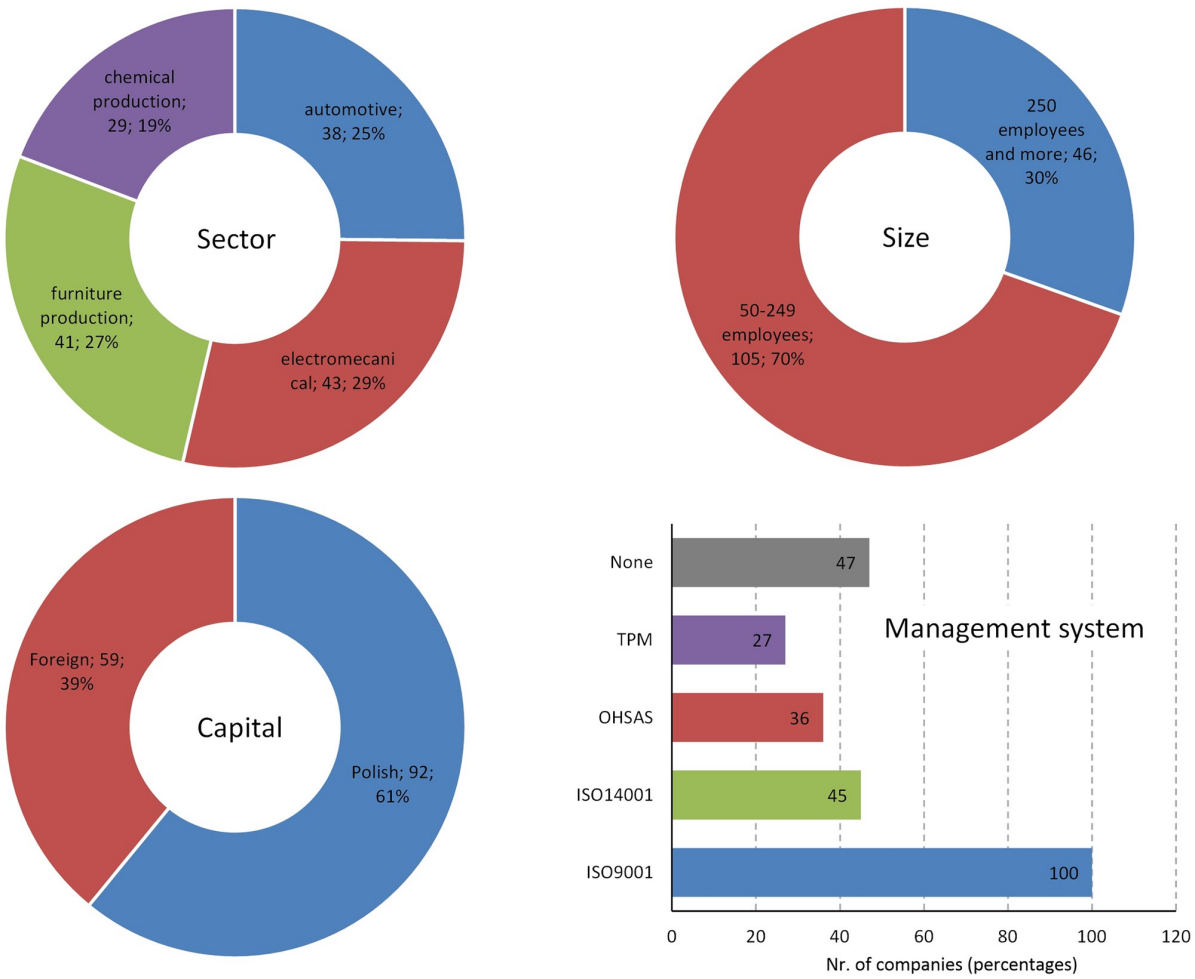
Basic characteristics of sample.

The survey collected 151 valid questionnaires. Incomplete and incorrectly completed questionnaires were excluded from the sample. The demographic characteristics of the companies involved are shown in Fig 1.

Non-response bias was examined by T-test. The samples were split into early (n = 83) and late (n = 68) groups. Within these two groups, ten randomly selected variables were tested (selected was: EnvPerf, SupFin, ProdInn, ProdIde, DocErr, EmePla, TechProb, MalaIS, DelFlex, CompResp). The results display insignificant differences (p < 0,05), indicating a lack of non-response bias. Scale reliability testing was used on 21 ordinal variables. Cronbach’s alpha reached 0.964. This is a very high value, which declares the scale’s reliability [78]. In the reliability test, the relevance of the examined variables was confirmed. The metrics of all 21 ordinal variables were validated by this test, as no potential increase in Cronbach’s alpha value after removal of the variables was demonstrated—the results are shown in Table 2.

**Table 2:** Testing of reliability if Item deleted.

Variable	Scale Mean if Item Deleted	Scale Variance if Item Deleted	Corrected Item-Total Correlation	Cronbach’s Alpha if Item Deleted
QualDef	43,8808	394,626	0,707	0,963
AsMist	44,1325	400,942	0,694	0,963
EnvPerf	44,7219	401,722	0,618	0,964
TimDel	43,4636	399,397	0,653	0,963
EmpQual	44,7219	395,295	0,781	0,962
SupFin	44,7947	397,538	0,743	0,962
AftSal	44,5960	393,482	0,803	0,962
ProdCap	44,0993	398,357	0,729	0,962
ProdInn	44,7152	396,658	0,758	0,962
ProdIde	44,7682	389,513	0,831	0,961
DocErr	44,3311	401,450	0,737	0,962
LonTim	43,9073	396,885	0,721	0,963
EmePla	44,3775	393,637	0,730	0,962
TechProb	44,5033	396,572	0,753	0,962
RaisPri	44,3709	388,955	0,789	0,962
SupInvRes	44,9735	404,279	0,716	0,963
MalaIS	44,7550	400,786	0,690	0,963
SupInvCos	44,8146	396,872	0,785	0,962
ComProb	44,6821	397,845	0,766	0,962
DelFlex	44,5430	397,250	0,733	0,962
CompResp	44,2252	393,829	0,734	0,962

### 4.1 Perception of risk factors

**Fig 2:**
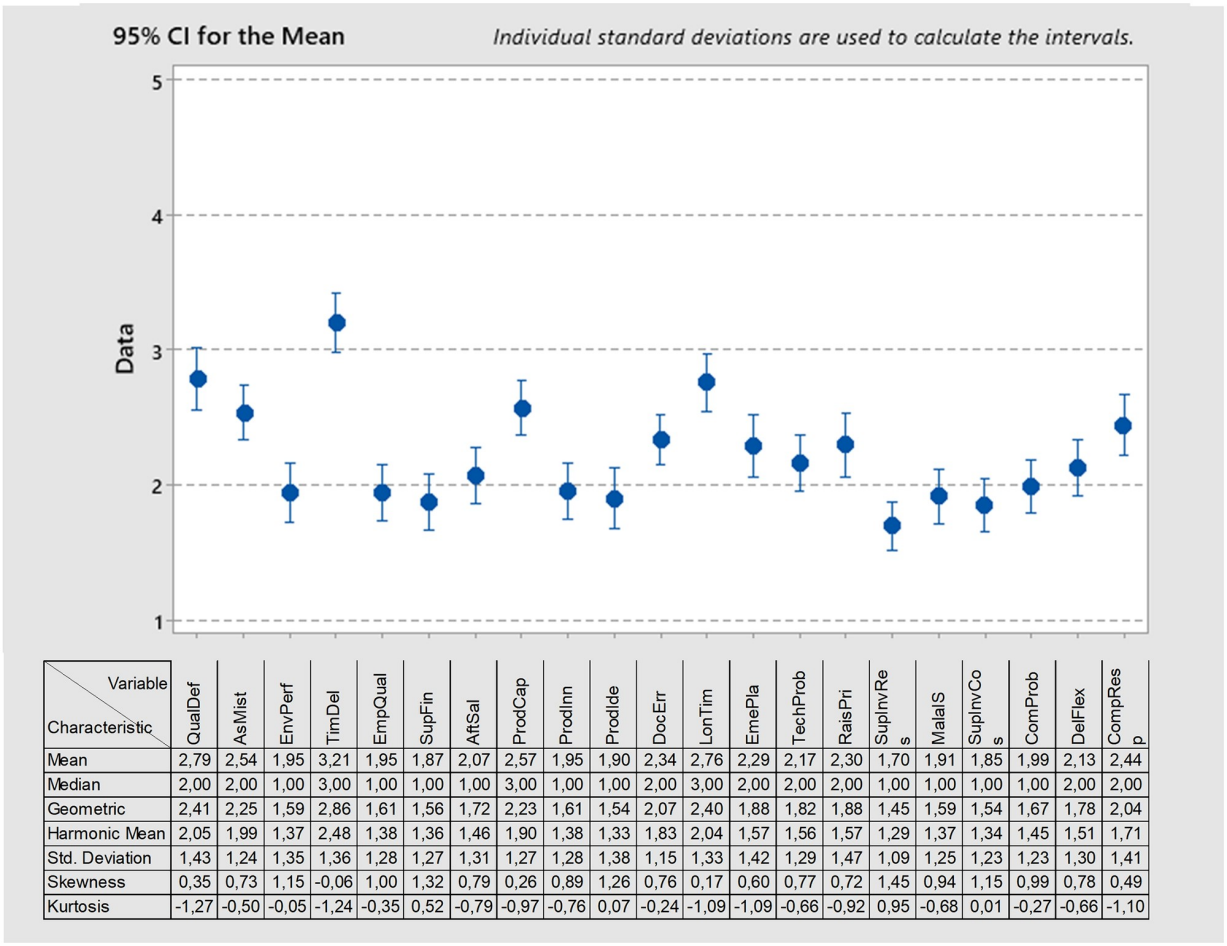
Perception of risk factors.

A set of 21 risk factors were to a basic statistical survey. To discover which risks are perceived by organizations as high and lower (5-point scale: higher value = higher risk). The results are presented in Fig 2. The figure contains basic metrics of descriptive statistics (e.g., mean) and an interval chart allowing for the interpretation of the average risk value and its reliability (within 95% CI). There was no statistically significant difference between the perception of risks between organizations that implemented one of the monitored management systems and organizations that did not. The results show that the three most serious risk factors include timely delivery (TimDel), quality defects of products (QualDef), and long order processing time (LonTim). On this basis, it can be concluded that for the surveyed companies, factors related to logistic aspects ensuring continuity of supplies and the possibility of just-in-time production, as well as issues related to the quality of products, are particularly important. All these factors significantly impact the client’s final satisfaction, which seems to be of key importance for the surveyed companies.

On the other hand, lower risk intensity was perceived by organizations for several types of risks. There are several that have reached a value of less than 2.0: low level of environmental performance (EnvPerf), low level of employee qualification (EmpQual), suppliers’ financial standing (SupFin), low level of product innovation (ProdInn), problem with product identification (ProdIde), low level of supplier involvement in joint research and development (SupInvRes), maladjustment of information systems in communication (MalaIS), low level of supplier involvement to reducing operating costs (SupInvCos) communication problems (ComProb). Therefore we can assume that the surveyed companies do not consider the risk factors related to the proper functioning of internal processes at suppliers, and the willingness to cooperate within the supply chain is essential. This interesting observation will be discussed in more detail in the discussion section.

### 4.2 Relationships between risk factors

**Fig 3:**
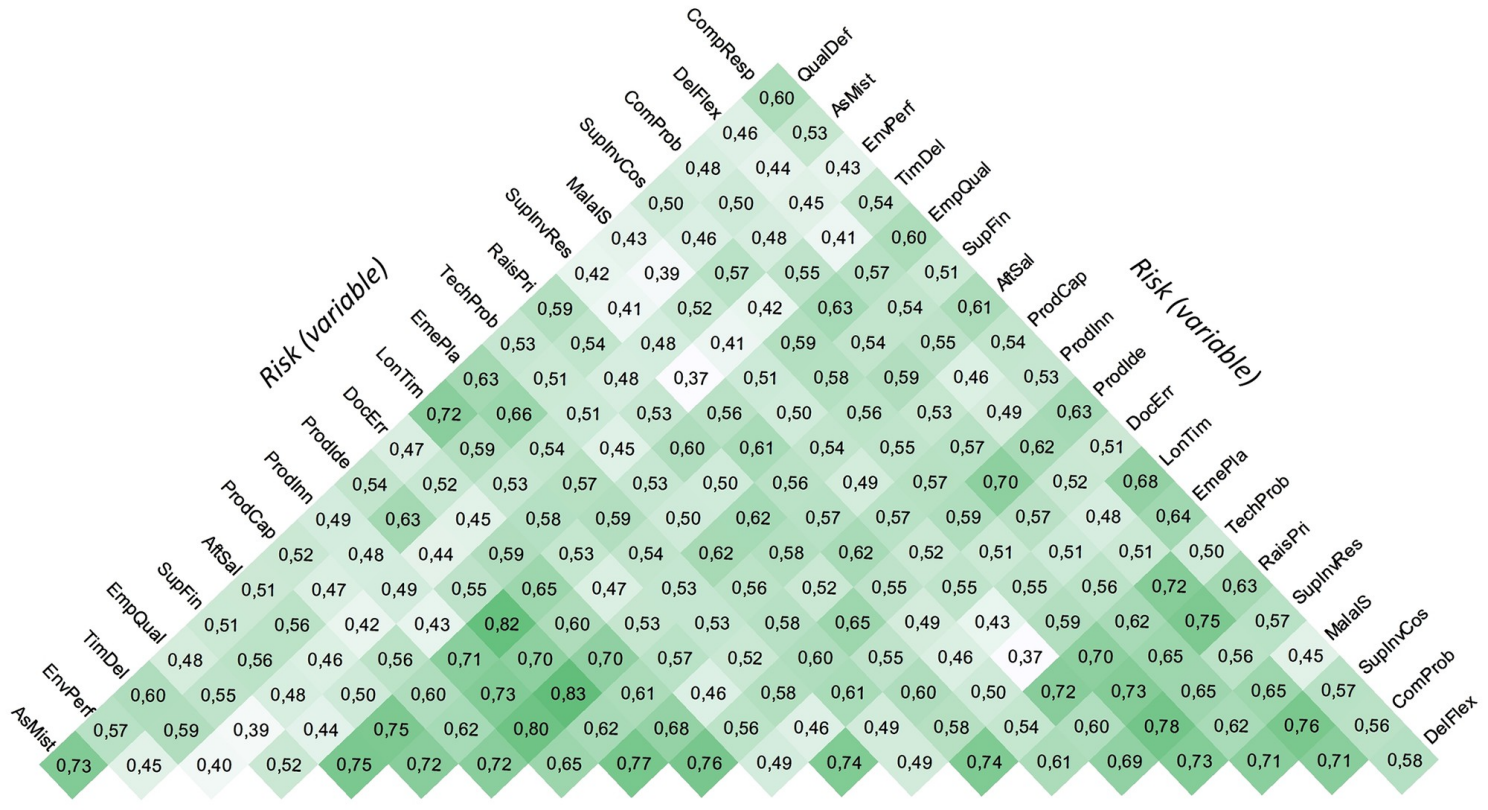
Correlation heatmap (values represents the Pearson linear correlation coefficient).

A bivariate correlation analysis examined the correlation structure between risk factors. The structure of the interrelationships was relatively complex, and the investigation identified numerous statistically significant relationships. It should be noted that only a positive correlation was identified between the variables. The size of the correlation coefficient ranged from 0.37 to 0.83. The intensity of the mutual relations is shown in Fig 3.

Since it can be seen that the structure of mutual relations is relatively complex, we can assume the existence of latent factors. To examine our assumption, we used factor analysis. Several basic measures tested the suitability of the data. The Kaiser-Meyer-Olkin Sampling Adequacy Measure reached 0.939 (the minimum recommended value is 0.700), and Bartlett’s Test of Sphericity reached Approx. Chi-Square at 2854.8 at a significance level of less than 0.001 (maximum recommended value is 0.05). At the same time, an analysis of communalities was conducted. This did not show the irrelevance of any variable, as the extraction values of the variables ranged from 0.496 to 0.843. This means that the data was sufficient to reduce the dimensions.

The analysis revealed three latent components among the 21 risk factors, which explain 71.675% of the variability of all risk factors–Table 3. When choosing the number of components, Kaiser’s rule was used as a base, and among the relevant components, only those had an initial eigenvalue value higher than 1,000. Table 4 contains the Rotated component matrix, and correlation coefficients between the variables and the individual components (latent variables). The matrix was rotated by the Varimax method with Kaiser normalization, with rotation converging in seven iterations. Significant cross-correlation was not identified for any variable.

**Table 3:** Factor analysis results—Total variance explained by principal component analysis.

Compo-nent	Initial Eigenvalues			Extraction Sums of Squared Loadings			Rotation Sums of Squared Loadings		
	Total	% of Var.	Cum. %	Total	% of Var.	Cum. %	Total	% of Var.	Cum. %
1	12,320	58,667	58,667	12,320	58,667	58,667	5,214	24,830	24,830
2	1,452	6,916	65,583	1,452	6,916	65,583	5,134	24,449	49,280
3	1,279	6,091	71,675	1,279	6,091	71,675	4,703	22,395	71,675
4	0,766	3,646	75,321						
…	…	…	…	…	…	…	…	…	…
21	0,099	0,470	100,000						

**Table 4:** Rotated component matrix.

Varible/Component	Component 1	Component 2	Component 3
AftSal	**0,795**	0,323	0,305
ProdInn	**0,792**	0,345	0,211
ProdIde	**0,770**	0,332	0,369
EmpQual	**0,755**	0,310	0,325
SupFin	**0,735**	0,310	0,282
DocErr	**0,700**	0,315	0,300
ProdCap	**0,619**	0,313	0,376
SupInvCos	0,280	**0,822**	0,297
MalaIS	0,309	**0,797**	0,136
DelFlex	0,274	**0,774**	0,268
TechProb	0,335	**0,709**	0,302
SupInvRes	0,400	**0,694**	0,190
RaisPri	0,297	**0,664**	0,452
ComProb	0,402	**0,638**	0,329
EnvPerf	0,165	**0,486**	0,483
QualDef	0,225	0,255	**0,810**
LonTim	0,226	0,293	**0,791**
EmePla	0,286	0,292	**0,749**
AsMist	0,373	0,168	**0,722**
TimDel	0,360	0,173	**0,664**
CompResp	0,359	0,374	**0,596**

The three components could be named based on the intensity of their relationships to specific risk factors. In naming, the internal meaning of risk factors was considered, and a search was done for common features of those risk factors that formed a group belonging to a specific component. The resulting components (meta-factors) are as follows:

- ***Component 1*: *Management system risks***–it is a component that consists mainly of variables such as Low level of after-sales service (AftSal), low level of product innovation (ProdInn), problem with product identification (ProdIde), low level of employee qualifications (EmpQual), Suppliers’ financial standing (SupFin), errors in the delivery documentation (DocErr), limited production capacity (ProdCap). These risks relate in some way directly or indirectly to the management system.
- ***Component 2*: *Environment risks***–this type of risk consists mainly of variables such as a low level of supplier involvement to reducing operating costs (SupInvCos), maladjustment of information systems in communication (MalaIS), low level of delivery flexibility (DelFlex), technological problems (TechProb), low level of supplier involvement in joint research and development (SupInvRes), unjustified raising prices for products (RaisPri), communication problems (ComProb), low level of environmental performance of products (EnvPerf). This type of risk is primarily external in nature and its source is often factors that are outside the organization’s environment.
- ***Component 3*: *Process risks***–risks of this type consist mainly of the following risk factors: quality defects of products (QualDef), long order processing time (LonTim), no emergency delivery plans (EmePla), assortment mistakes in deliveries (AsMist), threats to timely deliveries (TimDel), long response time to complaints (CompResp). All these risks are related to the process and its parameters.

**Fig 4:**
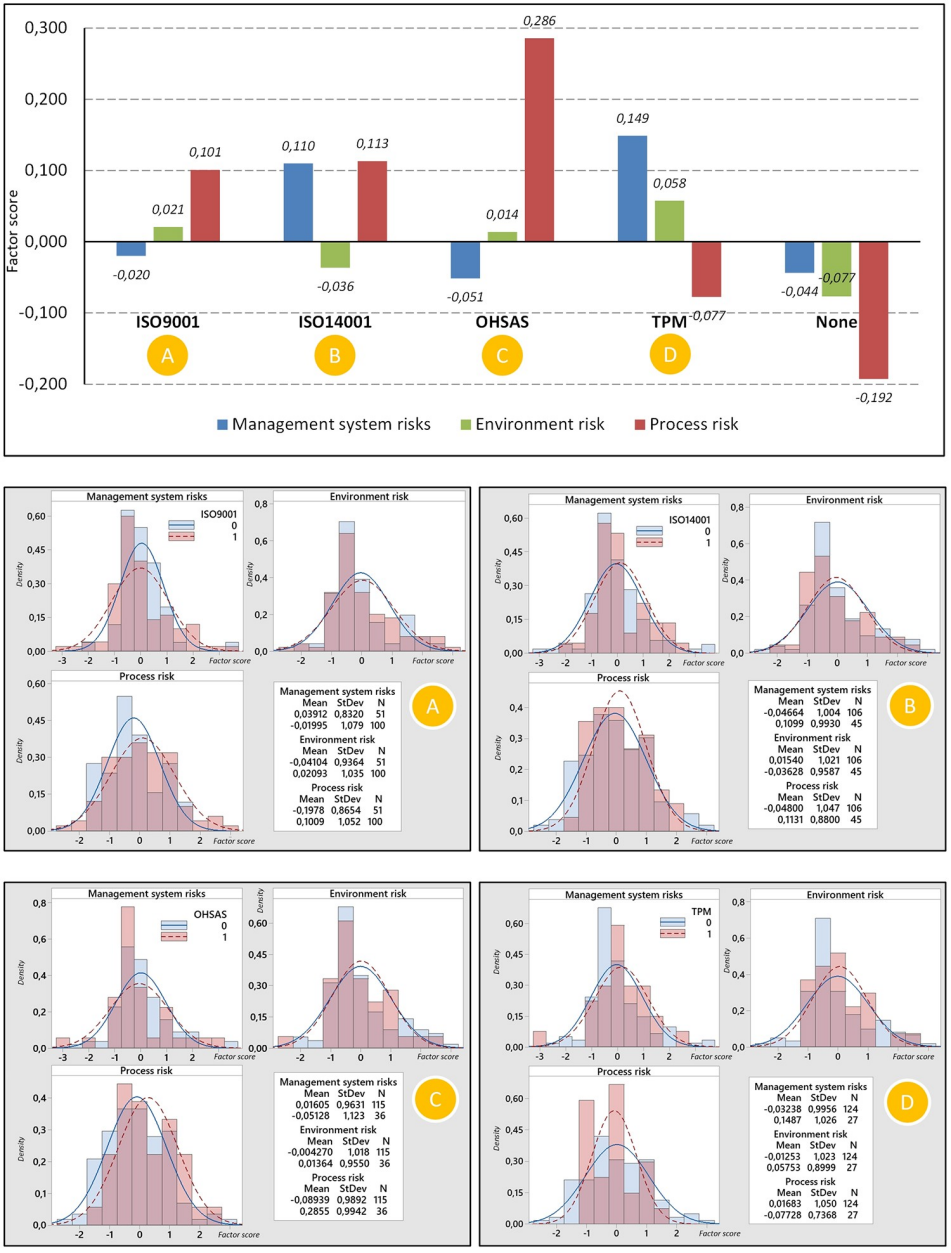
Relationships between particular management systems and three identified meta-factors of risks.

**Fig 5:**
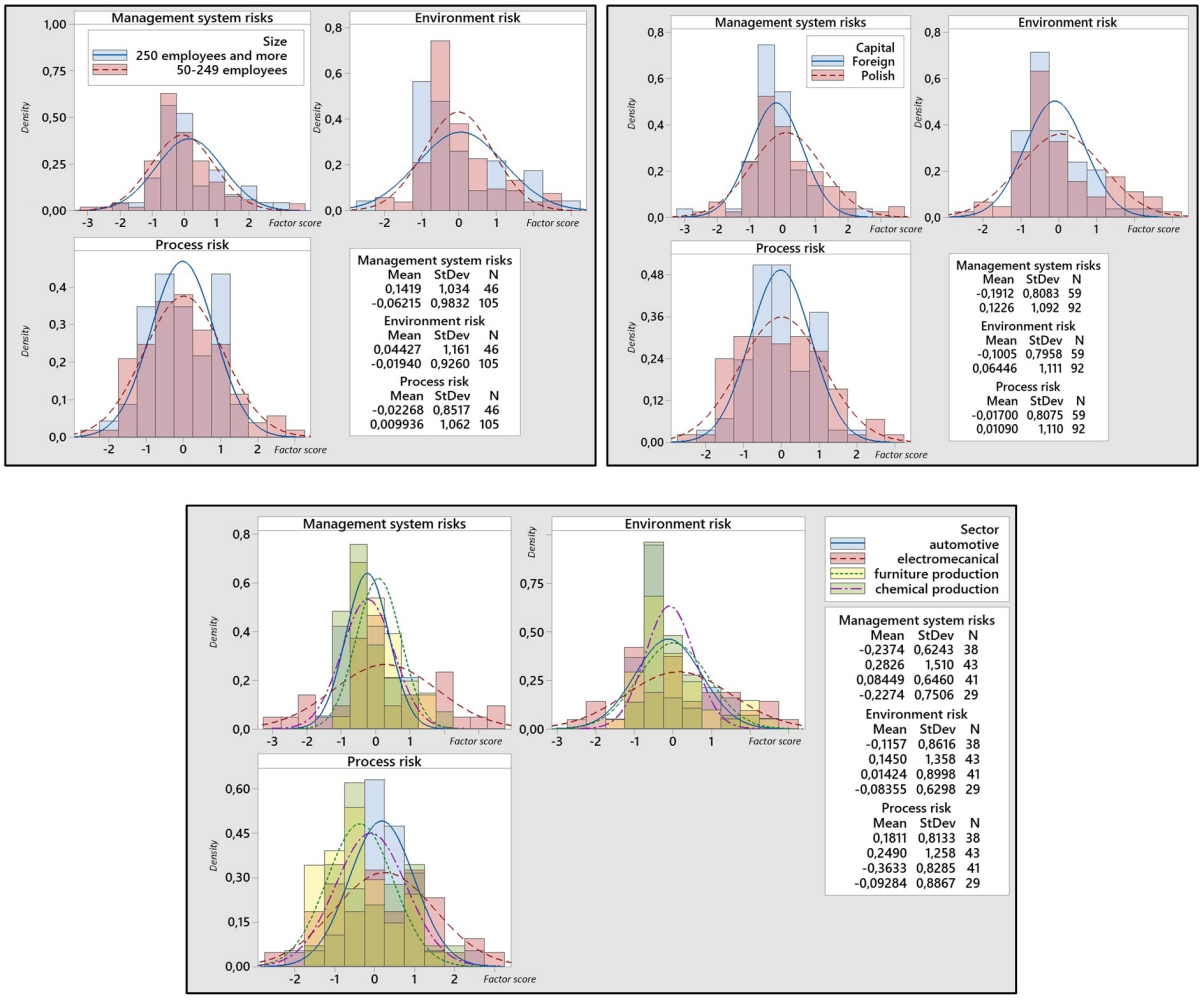
Relationships between size, capital and sector and three identified meta-factors of risks.

We have subjected the three identified components–management system risk, environmental risk, and process risk–to a deeper analysis. The goal was to determine whether the perception of these three meta-factors of risks differs depending on which management system the organizations implement and explore other potential relationships. For each organization, three new variables were recorded (one variable for each component). Values for individual organizations and specific components were calculated based on linear regression and were standardized to a Z-score. Fig 4 shows the results of the intensity of the three meta-factor of risks (components) divided according to the management system. Positive values of the meta-factor of risk mean that the organization attaches greater importance to it; negative values indicate lower importance. Fig 5 shows the other three analyzed variables—size, sector, and capital. Table 5 also contains the p-values of statistical testing differences for individual aspects that entered our research. The t-test was used in the analysis except for the sector variable, where an Anova test was used.

**Table 5:** Resulting p-values.

Variable/Component	Management system risks	Environmental risks	Process risk
ISO 9001	0,645	0,356	**0,033**
ISO 14001	0,190	0,384	0,167
OHSAS	0,627	0,538	**0,026**
TPS	0,204	0,361	0,706
Size	0,261	0,743	0,842
Capital	**0,045**	0,290	0,859
Sector (Anova)	**0,043**	0,716	**0,014**

Based on statistical analysis, it can be stated that several relationships were identified between the observed variables and meta-factors of risks (components). These findings are as follows:

- Finding 1: Organizations with QMS give more importance to process risks than organizations without QMS
- Finding 2: Organizations with H&SMS give more importance to process risks than organizations without these systems
- Finding 3: Organizations with foreign capital given to management system risks less importance than organizations with capital from Poland
- Finding 4: Organizations operating in different sectors perceive management systems’ risks and process risk differently
- Finding 5: The importance of environmental risks is approximately the same for all organizations, regardless of whether they have a management system implemented, and also irrespective of their size, capital, and sector.

In Fig 4, we can see the intensity of the three meta-factors of risk about the implemented management system. It should be remembered that these meta-factors are not correlated with each other.

For organizations implementing QMS (e.g. ISO 9001), expectations are primarily reflected in higher demands for process risk. This is not a surprise as, according to the requirements of standardized management systems (e.g., QMS), enterprises should consider process risk in strategic and operational planning and take actions to eliminate the risk. It is also interesting to note that management system risk is not among the threats from suppliers to companies implementing QMS. Most organizations with a QMS require the same management system from their suppliers, which would explain the low expected risks associated with this meta-factor. Organizations with QMS also perceive a low level of Environment risks concerning suppliers. It is also worth paying attention to the fact that enterprises implementing the requirements of ISO 9001 are slightly interested in systemic risk. The specific distribution of factor scores for all three meta-factors for organizations with QMS can be seen in part A of Fig 4.

Organizations with EMS (e.g., ISO 14001) give more importance to Management system risk and Process risk. EMS is often closely linked with QMS and, together with other management systems, forms an integrated management system. The integrated management system has the task of covering various types of requirements through the processes that take place in the organization. Emphasis on these meta-factors would therefore be justified. Environment risk (not environmental risk) is less important than the two mentioned. The specific distribution of factor scores for all three meta-factors for organizations with QMS can be seen in part B of Fig 4.

If we look at part C of Fig 4, we can see that organizations implementing H&SMS (e.g., OHSAS) give much more importance to Process risk. This result explains the purpose of H&SMS systems quite well—namely, eliminating risks associated with work and the working environment. According to some sources, this type of risk often appears in processes [79].

Part D of Fig 4 deals with analyzing the importance of risk factors in implementing the Toyota Production System. In organizations with such a system, no statistically significant differences were found in the perception of the importance of the three identified factors. The reason may be that the Toyota Production System is a long-established approach to the complex management of manufacturing companies, and the focus on individual aspects is generally balanced [80].

When analyzing the data in Figs 4 and 5, it can be concluded that organizations with standardized management systems pay attention to process risk management. This is not a surprise as, according to the requirements of standardized management systems (e.g., QMS), enterprises should consider process risk in strategic and operational planning and take actions to eliminate the risk. It is also worth paying attention to the fact that enterprises implementing the requirements of ISO 9001 are slightly interested in systemic risk. In contrast, organizations that have EMS have environmental risks. Environmental risk-shaping factors are perceived similarly (usually the average importance of these aspects), regardless of the implemented management system, industry, or company profile. Respondents who have implemented TPS requirements, in turn, put the greatest emphasis on the factors shaping systemic risk. Taking into account other factors, it can be concluded that enterprises with foreign capital attach less importance to systemic risk than enterprises with Polish capital. Moreover, enterprises from various industries have different approaches to assessing the importance of individual risk groups.

## 5. Discussion

Er Kara and Oktay Fırat [81] recognize that the procurement process is a critical function that is strategically important to the company’s success. For this reason, it is essential to correctly identify risk factors that may occur as part of the cooperation between the supplier and the recipient, their assessment, and implementation of improvement actions. The article presents a risk assessment and the risk requirements that enterprises pose to their suppliers. The novelty presented in the article was to establish how the implementation of the requirements of management systems such as QMS, EMS, H&SMS, and TPS translates into the perception of risk towards suppliers. The conducted research process allowed for drawing some interesting conclusions. It was found that risk factors related to logistic aspects ensuring continuity of supplies and affecting the quality of finished products are particularly important for the surveyed companies. This is understandable because companies are primarily concerned about possible delivery delays that may disrupt the production process and the need to maintain a high inventory level [82]. Timeliness and flexibility of deliveries are critical criteria for supplier evaluation. Timeliness of deliveries is essential when manufacturers use the just-in-time concept [83].

Delays in the timeliness of deliveries may, in the case of enterprises from industrial sectors, cause interruptions in production processes. For this reason, providers are increasingly expected to develop business continuity plans and disaster recovery plans, especially in an emergency [84–87]. Attention to the quality of semi-finished products is also not surprising because, as Yoo [88] rightly identifies, problems with the quality of finished products are the main factor of product returns and customer loss. Product quality is most often measured by the level of defective deliveries—a percentage ratio of the number of defective products delivered to the total number of products delivered. In the case of mass purchases, the quality of deliveries is measured using the Defective Parts Per Million index [89, 90]. In the case of production sectors, the acceptable value of this indicator is determined [91]. A high value of this ratio may eliminate the supplier from further cooperation [92]. However, it is quite surprising that the surveyed companies do not pay much attention to risk factors related to the proper functioning of internal processes at suppliers. This can be considered on two levels. First of all, enterprises, through a correct process of selecting suppliers, periodic assessments, and conducting audits, can ensure that the effective implementation of key processes by suppliers is not endangered. In case of doubts, they can implement corrective actions on an ongoing basis. On the other hand, it can be stated that some manufacturing companies do not pay much attention to internal processes at suppliers if they ensure timely delivery of components that meet the adopted quality standards [93]. Further research allowed for the possibility to state that external risk factors do not belong to the least significant group for the surveyed companies (no matter which management system they implemented), so it can be assumed that the first level is closer to the truth.

To deepen the research process, the risk was classified into three main groups: management system risks, environment risks, and process risks. This allowed for the formulation of some original conclusions. First of all, it is puzzling that companies that comply with H&SMS (OHSAS 18001/ISO 45001) standards have the highest requirements regarding process risk. Admittedly, this standard requires the organization to identify hazards and assess occupational health and safety risks related to the activities and services conducted, define the necessary supervision measures, and set clear goals to improve the effects of activities in the area of health and safety [94]. However, higher requirements of enterprises complying with the requirements of this standard than ISO 9001 may come as a surprise, especially since the ISO 9001 standard requires suppliers to ensure the correct implementation of key processes [95].

Secondly, it is worth emphasizing that organizations adhering to the requirements of ISO 9001 attach the lowest importance to systemic risk, while enterprises complying with TPS requirements—are the highest. While the high level of interest of enterprises using TPS guidelines in systemic risk is understandable since this system is based on the flow of information and close cooperation with suppliers [80], the low level of interest on the part of enterprises implementing the requirements of ISO 9001 is somewhat surprising. after all, this system places a strong emphasis on the correct implementation of key management processes in the organization, cooperation with suppliers and the external environment [96].

Thirdly, organizations that comply with the requirements of ISO 14001 have the lowest requirements for suppliers in the area of external (environmental) risk. This is an interesting observation, as the ISO 14001 standard is generally associated with ecological issues and is in line with sustainable development. However, it is less concerned with communication problems or fluctuations in commodity prices.

Fourthly, enterprises that have not decided to implement the analyzed management systems treat risk management issues superficially. Their requirements towards suppliers are particularly low in the area of process risk. Therefore, it can be concluded that standardized management systems that include risk management requirements mobilize enterprises to take this important issue more seriously [26].

Fifthly, enterprises representing various sectors assess the rank of risk differently. Therefore it can be concluded that the specificity of the industry and the company’s conditions affect the process of analyzing and assessing the risk of cooperation with supplies.

### 5.1 Theoretical and practical implications

Scientific representatives will learn about the impact of implementing management systems on the perception of risk, which is defined as a situation of uncertainty that may occur in the future. The main findings of the article can be considered a valuable contribution to the discussion on the importance of implementing standardized management systems in the context of cooperation with suppliers and more broadly within the supply chain, i.e., in an area that seems to be still insufficiently researched in the literature [26, 97].

The research expands the knowledge on the impact of management systems on cooperation with suppliers within the supply chain. The research results can be used by managers who manage supply chains to include in the management strategy risk factors that are downplayed or ignored by manufacturing companies and to pay special attention to factors considered critical. Research shows that risk factors ensuring continuity of supplies and the expected quality of products are particularly important. Entrepreneurs should consider introducing preventive measures in this area (e.g., maintaining an optimal level of stocks, signing contracts with backup suppliers, etc.). There is also a need to emphasize the external risk factors of cooperation with suppliers.

From the engineering point of view, this study has several implications. Current research points to an increasing need to manage risks associated with supply chain management [98]. Our research has shown that the perception of risks is not at the same level in companies with regard to the implemented management system. A greater emphasis on the types of risks that have been overlooked so far (e.g., Environment risk) would contribute to the growth of supply chain resilience [98], or, as current research suggests, could help better determine the occurrence of operational risk events [99, 100].

### 5.2 Research limitations

As with many other pieces of research, some limitations can be addressed in future research. This study was conducted in Poland, and the results represent the views of Polish companies. The generalizations outlined in the results and the discussion section may not apply to other countries, especially considering the country’s maturity and standardized management systems. A cross-cultural study identifying the differences in risk assessments could bring new context to this topic.

The second limitation is the sample size. In the research, the results of 151 organizations were processed, which represents a medium sample size. The sample size has a medium detection capability for statistical deviation analysis or statistical hypothesis testing. This means that medium and large deviations in the monitored areas under the influence of the sample size could be identified. Minor deviations may not have been observed, which may be a limitation of this study and the subject of the subsequent investigation. On the other hand, it should be noted that this study is exploratory, so it aims to explore a new area—risk factors in a new systematic way. In previous research designed this way, a smaller sample is usually sufficient, as seen in similarly focused studies [101–103].

The third limitation may be the identification of risk factors. Although an extensive literature review was undertaken at the beginning of this study, which should ensure the validity of risk factors, there may still be a possibility that a risk factor was not included in the study. Finally, the context of the Covid-19 pandemic suggests that the structure of risk factors may not be constant over time. Further studies can further expand the knowledge regarding other risk factors.

## 6. Summary

Risk management is one of the essential components of building a strategy in today’s turbulent environment. This fact is noticed by the authors of standardized management systems, who strongly emphasize risk management in the latest editions of standards. The research aimed to fill the gap in the literature on the subject and cover the attitude of manufacturing companies to supplier assessment from the perspective of risk analysis, taking into account the impact of the implementation of management systems. The research results showed that companies implementing standardized management systems take the issue of risk analysis and management more seriously than organizations that do not implement such systems. The research also highlighted the differences in the perception and assessment of risk caused by implementing various management systems. The study also found that the industry and business profile specificity also affect the risk assessment in cooperation with suppliers.

Although necessary, this research can be developed in several respects. First, it would be quite interesting to repeat the research process on a group of organizations that have implemented management systems. Secondly, an interesting idea would also be to investigate how suppliers assess companies’ involvement in risk management due to the management systems implemented by these companies. Thirdly, these studies were conducted in the pre-pandemic period. It is also worth repeating in a period in which there are additional barriers and difficulties related to the COVID-19 pandemic.

## Supporting information

S1 Data (XLSX)
